# Prognostic Value of Albumin to D-Dimer Ratio in Advanced Gastric Cancer

**DOI:** 10.1155/2021/9973743

**Published:** 2021-06-21

**Authors:** Liqun Zhang, Zhuo Wang, Jiawen Xiao, Zhiyan Zhang, Haijing Li, Fang Li, Lisha Zhang, Yuanhe Wang

**Affiliations:** ^1^Department of Medical Oncology, Shenyang Fifth People Hospital, Tiexi District, Shenyang 110020, Liaoning Province, China; ^2^Department of Medical Oncology, Liaohua Hospital, Hongwei District, Liaoyang 111003, Liaoning Province, China; ^3^Department of Hepatobiliary Surgery, Liaoning Cancer Hospital & Institute, Cancer Hospital of China Medical University, No. 44 Xiaoheyan Road, Dadong District, Shenyang 110042, Liaoning Province, China; ^4^Department of Gastrointestinal Surgery, The Second Hospital Affiliated to Harbin Medical University, No. 246, Xuefu Road, Nangang District, Harbin 150086, Heilongjiang Province, China; ^5^Medical Oncology Department of Gastrointestinal Cancer, Liaoning Cancer Hospital & Institute, Cancer Hospital of China Medical University, No. 44 Xiaoheyan Road, Dadong District, Shenyang 110042, Liaoning Province, China

## Abstract

Gastric cancer (GC) is one of the most common malignancies worldwide. Notably, patients with advanced GC have a poor prognosis and quality of life, prompting the need for further studies on its prognostic markers. Among these, albumin and D-dimer are often used as prognostic factors in the prediction of a variety of tumors. Moreover, the albumin to D-dimer ratio (ADR) may be an improved predictor of chemotherapy effect and survival compared to albumin and D-dimer alone, but few studies have investigated this issue. Thus, we explored the relationship between pretreatment ADR and prognosis in advanced GC treated with first-line chemotherapy. A total of 247 advanced unresectable GC patients treated with first-line chemotherapy were retrospectively included. The cut-off value for ADR was determined using the receiver operating characteristic (ROC) curve. The ADR had a cut-off value of 41.64. Compared to albumin and D-dimer alone, ADR had the highest area under curve (AUC) value (AUC = 0.730), followed by albumin (AUC = 0.659) and D-dimer (AUC = 0.719). Additionally, we found that patients with a low ADR (<41.64) had a lower disease control rate (77.9% vs. 92.5%, *P* < 0.01), shorter overall survival (OS) (271 vs. 389 days), and shorter progression-free survival (PFS) (118 vs. 192 days) than patients with a high ADR (≥41.64). Similar results were also found on subgroup analysis, and ADR was found to be an independent advanced GC prognostic factor on multivariate analysis (all *P* < 0.001). Low ADR was found to be correlated with poor therapeutic effects of chemotherapy and shortened OS and PFS. Therefore, pretreatment ADR may be a useful tool for predicting the effect of chemotherapy and prognosis in advanced GC patients treated with first-line chemotherapy.

## 1. Introduction

In 2017, around 1.2 million new gastric cancer (GC) cases and 865,000 stomach cancer-related deaths were recorded worldwide. In fact, it is speculated that 1 in 78 women and 1 in 33 men would develop GC over their lifetime [1]. Although GC incidence has declined in recent years, it is still one of the most common malignancies worldwide. Among its management options, surgery remains the primary curative modality for early GC, showing an excellent prognosis for early GC patients who underwent radical resection [2]. However, for advanced GC patients, the postoperative recurrence rate of simple surgical treatment is as high as 50%–70% due to a high risk of recurrence and metastasis [3]. For these cases, chemotherapy is one of its leading treatments, but its efficacy remains unsatisfactory, with a median survival time of only 6–13 months for patients receiving chemotherapy [4]. Considering all this, advanced GC poses a formidable challenge to patient survival. At present, the ideal prognostic indicator of early GC is still TNM staging, but for advanced GC patients, survival time is different despite following the same TNM staging. Thus, it is important to identify prognostic indicators and therapeutic predictive factors for advanced GC patients to be able to help doctors optimize treatment and improve patient survival.

Systemic inflammation and malnutrition are common comorbid conditions in patients with tumors [5, 6], wherein these conditions can cause tumor growth, tumor cell dissemination, and drug resistance, which all lead to a short survival time [7–9]. In our previous study, we found that sodium to globulin ratio was a novel and promising prognostic factor for GC patients [10]. In the present study, albumin, a potent protein produced by liver cells, has been widely used as a serum inflammatory and nutritional marker to predict mortality in critically ill patients [11, 12]. Moreover, the association between albumin level and cancer has been extensively studied, in which a series of studies have found that albumin acts as a prognostic factor and can predict clinical outcomes of hepatocellular carcinoma [13], prostate cancer [14], acute myeloblastic leukemia [15], and GC [16].

Furthermore, coagulation abnormalities are commonly found in cancer patients. Notably, the hypercoagulable state of patients with malignant tumors is considered to be related to tumor angiogenesis, growth, and dispersion, as well as metastatic cancer, ultimately leading to a poor prognosis [17]. In response to this, D-dimer has been found to reflect fibrinolysis and coagulation cascade activation [18]. In fact, the relationship between tumors and D-dimer has also gained attention in recent years, with studies showing its utility as a prognostic marker for the indication of tumor progression in colorectal [19], liver [20], lung [21], and gastric cancer patients [22].

Based on the aforementioned researches, albumin and D-dimer levels alone may be used as potential prognostic factors for GC. Decreases in albumin levels and increases in D-dimer levels may reflect the presence of high inflammation levels, malnutrition, and a hypercoagulable state. Moreover, combining both D-dimer and albumin, known as the albumin to D-dimer ratio (ADR), may reflect the inflammation, nutrition, and coagulation function of cancer patients at the same time, thereby improving the predictive accuracy for GC patient prognostication as compared to albumin and D-dimer alone. Interestingly, there have been studies investigating the combination of D-dimer and albumin for the prediction of survival prognosis in patients. Liu et al., for one, found that a combination of preoperative plasma D-dimer and serum albumin levels was notably associated with postoperative survival of esophageal squamous cell carcinoma patients treated with transthoracic esophagectomy [23]. Another study by He et al. also recently demonstrated that the combination of D-dimer and albumin may serve as a predictor of overall survival (OS) and distant metastasis-free survival in nasopharyngeal carcinoma patients [24].

Despite these findings, there are only a few studies on ADR as a valuable and novel prognostic marker in GC patients. Therefore, the aim of this study was to investigate the relationship between pretreatment ADR and prognosis in advanced GC patients treated with first-line chemotherapy.

## 2. Materials and Methods

### 2.1. Patients

We retrospectively reviewed a database of advanced GC patients treated with first-line chemotherapy, which was recorded at the Cancer Hospital of China Medical University between June 2014 and January 2019 (Shenyang, Liaoning, China). Ethical approval was obtained from the Ethical Committee of the Cancer Hospital of China Medical University prior to data collection. The inclusion criteria for this study were as follows: (1) patients who were histologically diagnosed as having advanced GC and had undergone no previous antitumor therapy; (2) patients who were diagnosed as Stage III or IV; (3) patients who were treated with first-line chemotherapy and were available for plasma albumin and D-dimer measurements before first-line chemotherapy. Meanwhile, the exclusion criteria for the final analysis were as follows: (1) incomplete data; (2) treatment with accepted anti-inflammatory and anticoagulant drugs before chemotherapy; (3) severe kidney and liver dysfunction; (4) infection, myocardial infarction, and thrombosis.

### 2.2. Data Collection

A total of 247 patients were finally included in the study. Hospital electronic records and patient notes were reviewed, including blood indexes before first-line chemotherapy, chemotherapy response, OS, and progression-free survival (PFS). Moreover, we defined the ratio of albumin (g/L) to D-dimer (mg/L) as ADR.

Chemotherapy efficacy was assessed after every 2 cycles using RECIST 1.1. Patients who failed first-line chemotherapy were followed up until they were lost to follow-up or had passed away, with the latest follow-up date in April 2020. Disease control rate (DCR) was defined as stable disease (SD), partial response (PR), and complete response (CR), wherein overall response rates (ORRs) included PR and CR. Additionally, PFS and OS were defined as the time from the beginning of first-line chemotherapy until disease progression (PFS) or last follow-up and death from any cause (OS).

### 2.3. Statistical Analysis

Data were analyzed using SPSS (IBM SPSS 23.0, SPSS Inc.) and GraphPad Prism (GraphPad Prism 8.0, USA), with all data expressed as medians (25th percentile and 75th percentile) (for data with skewed distribution). Receiver operating characteristic (ROC) curves were also used to calculate the ADR cut-off, and the Mann–Whitney *U* and chi-square tests were also used as appropriate. Univariate and multivariate analyses were performed, and survival analysis was performed using Kaplan-Meier and log-rank tests. Statistical significance was set at *P* < 0.05 for all analyses.

## 3. Results

### 3.1. ROC Curve Analysis

**Figure 1:**
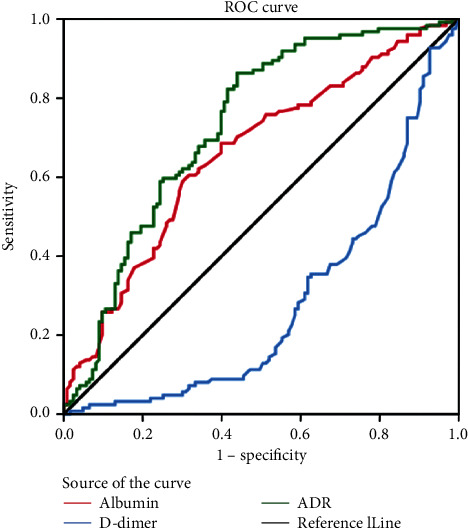
Receiver operating characteristic curves for the ability of pretreatment ADR, albumin, and D-dimer to predict mortality for advanced gastric cancer patients. ADR, albumin to D-dimer ratio.

The cut-off values of pretreatment ADR, albumin, and D-dimer for predicting mortality were determined by ROC curve analysis, with an optimal ADR cut-off value determined at 41.64 [area under the curve (AUC) = 0.730 (*P* < 0.001)]. Meanwhile, optimal cut-off values of albumin and D-dimer were noted to be 40.55 (AUC = 0.659, *P* < 0.001) and 0.93 (AUC = 0.719, *P* < 0.001), respectively. Overall, ADR had a higher AUC value than albumin and D-dimer, implying better reliability and prediction performance of ADR as a prediction model for predicting prognosis. Based on the ADR cut-off value, we then divided patients into two groups in the following research (Figure 1).

### 3.2. Relationship between the ADR and Clinicopathological Factors

Clinical characteristics of the patients are shown in Table 1. The median age was 59 years (range: 52–64 years), BMI was 21.51 kg/m^2^ (range: 19.57–23.43 kg/m^2^), and the majority of patients (66.0%) were men. The baseline ECOG performance status was Grades 0–1 in 84.6% of patients, and pathological staging was poorly differentiated in 180 patients (72.9%). Specifically, patients with 2 or more distant organ metastases, who had peritoneal metastasis, and who were diagnosed as having Stage IV cancer accounted for 35.2%, 30.4%, and 79.4% of patients, respectively.

**Figure 2:**
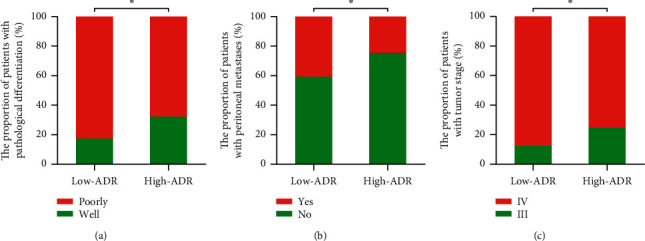
Relationship between the pretreatment ADR and (a) pathological differentiation, (b) peritoneal metastasis, and (c) tumor stage. ^*∗*^*P* < 0.05; ADR, albumin to D-dimer ratio.

**Figure 3:**
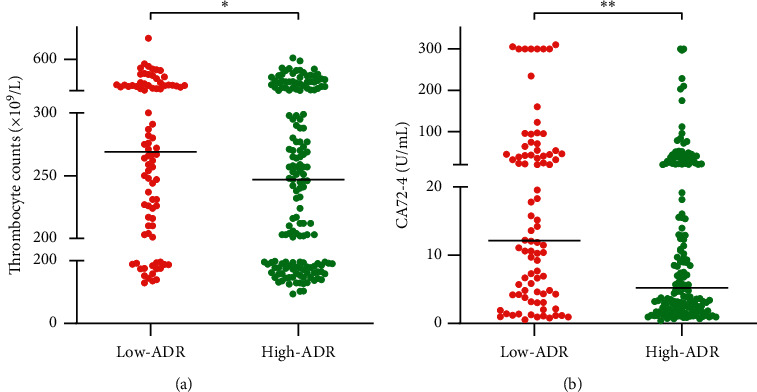
Relationship between the pretreatment ADR and (a) thrombocyte counts and (b) CA72-4 level. ^*∗*^*P* < 0.05; ^*∗∗*^*P* < 0.01; ADR, albumin to D-dimer ratio; CA72-4, carbohydrate antigen 72-4.

We also analyzed the association between pretreatment ADR and clinicopathological parameters (Table 1 and Figures 2 and 3). The results revealed that the low-ADR group (ADR < 41.64) had higher thrombocyte counts (median thrombocyte count [×10^9^/L], 269 vs. 247, *P*=0.031) and higher carbohydrate antigen (CA) 72–4 levels (median CA72-4 [U/mL], 12.13 vs. 5.19, *P*=0.001) and had more occurrences of poorly differentiated adenocarcinoma (82.6% vs. 67.7%, *P*=0.012), Stage IV disease (87.2% vs. 75.2%, *P*=0.026), and peritoneal metastasis (40.7% vs. 24.8%, *P*=0.010) as compared to the high-ADR group (ADR ≥ 41.64).

### 3.3. Relationship between the ADR and Effect of First-Line Chemotherapy

**Figure 4:**
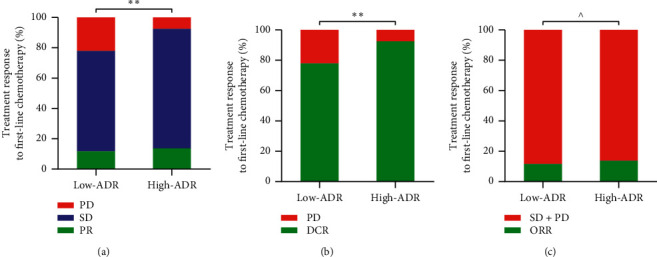
Relationship between the pretreatment ADR and (a) PD, SD, PR, (b) DCR, and (c) ORR. ^*∗∗*^*P* < 0.01; ^*P* > 0.05; ADR, albumin to D-dimer ratio; PD, progressive disease; SD, stable disease; PR, partial response; DCR, disease control rate; ORR, objective response rate.

The relationship between ADR and treatment effect is also shown in Figure 4, showing that no patients achieved CR following the standard criteria. High-ADR patients achieved a lower proportion of PD (7.5% vs. 22.1%) and a higher proportion of PR (13.7% vs. 11.6%) and SD (78.8% vs. 66.3%) as compared to low-ADR patients (*P* < 0.01). During first-line treatment, DCR was found to be 92.5% in high-ADR patients and 77.9% in low-ADR patients. Moreover, the ORR was 13.7% in the high-ADR group and 11.6% in the low-ADR group, but the difference was not statistically significant (*P* > 0.05).

### 3.4. Analysis of the Survival Stratified by Patient and Tumor Characteristics

Tables 2 and 3 show the results of the univariate and multivariate analyses. Univariate analysis revealed that PFS in GC patients was significantly associated with CA72-4 (*P*=0.003), distant organ metastasis (*P*=0.002), TNM staging (*P*=0.044), peritoneal metastasis (*P*=0.004), and ADR (*P* < 0.001). Additionally, the OS in GC patients was associated with age (*P*=0.004), pathological differentiation (*P*=0.017), distant organ metastasis (*P*=0.003), peritoneal metastasis (*P* < 0.001), and ADR (*P* < 0.001). On the other hand, multivariate analyses revealed that ADR independently predicted PFS in GC patients (hazard ratio [HR] = 0.509, *P* < 0.001), which was also the dominant independent prognostic factor for OS (HR = 0.317, *P* < 0.001).

### 3.5. Association between ADR and Clinical Outcome

**Figure 5:**
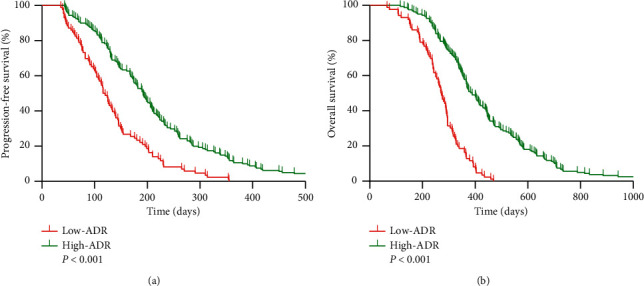
Kaplan–Meier curves of (a) progression-free survival and (b) overall survival based on the ADR cut-off value in patients. ADR, albumin to D-dimer ratio.

Median OS and PFS were noted to be 337 and 167 days, respectively, in the overall population. Survival analysis further showed that high-ADR patients achieved better median OS (389 vs. 271 days, *P* < 0.001) and median PFS (192 vs. 118 days, *P* < 0.001) as compared to low-ADR patients (Figure 5).

**Figure 6:**
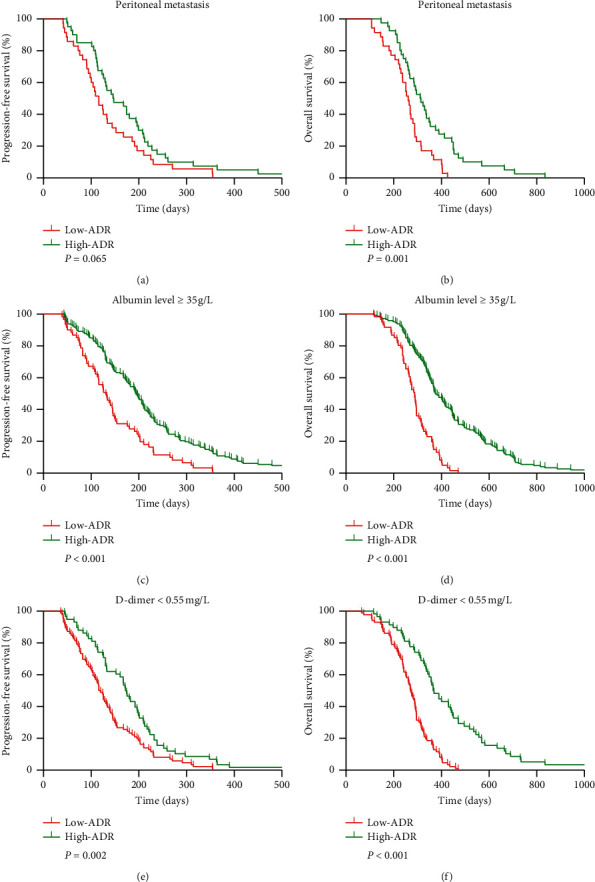
Subgroup analysis for the association between progression-free survival and overall survival and ADR in the subgroups stratified by (a, b) peritoneal metastasis, (c, d) normal albumin level, and (e, f) normal D-dimer level. ADR, albumin to D-dimer ratio.

A subgroup analysis was conducted to compare the survival between low-ADR and high-ADR groups in terms of entity popularity (Figure 6). In the normal albumin level (≥35 g/L) and normal D-dimer level (<0.55 mg/L) groups, high-ADR patients had better median OS (normal albumin level: 376 vs. 288 days, *P* < 0.001; normal D-dimer level: 366 vs. 271 days, *P* < 0.001) and median PFS (normal albumin level: 194 vs. 131 days, *P* < 0.001; normal D-dimer level: 173 vs. 118 days, *P*=0.002) as compared to low-ADR patients. In the peritoneal metastasis subgroup, ADR remained an indicator of OS (median OS, 313 vs. 261 days, *P*=0.001). Furthermore, there was a trend toward improved PFS (median PFS, 147 vs. 116 days) in high-ADR patients compared to that in low-ADR patients in the peritoneal metastasis subgroup, but the difference was not statistically significant (*P*=0.065).

## 4. Discussion

Inflammation, malnutrition, and dysfunction of the coagulation system are often observed in GC [25–27]. Previous research has shown that high levels of proinflammatory cytokines, poor nutritional status, and a hypercoagulable state in GC patients are correlated with poor prognosis [28–30]. In that regard, lower albumin levels, as a marker of inflammatory and nutritional status, are associated with poor prognosis in many aggressive tumors [31, 32]. On the other hand, elevated D-dimer levels, as a surrogate marker of hypercoagulability and inflammatory status, correlated with poor survival in a variety of cancers [18, 33, 34]. Combining both markers, the albumin to D-dimer ratio (ADR) may reflect the overall inflammation, nutrition, and blood coagulation situation of cancer patients simultaneously, with improved accuracy compared to albumin and D-dimer alone; however, only a few data on this subject are available.

To date and to the best of our knowledge, this study is the first to report the association of pretreatment ADR with response to therapy and prognosis in advanced GC patients receiving first-line chemotherapy. ROC curve analysis was performed to identify the cut-off value, with the results showing that the AUC was higher for ADR than for both albumin and D-dimer in detecting patient OS. Thus, ADR may be a more accurate prognostic predictor than albumin and D-dimer alone. Subsequently, the patients were divided into high-ADR and low-ADR groups based on the ADR cut-off points for further analysis. We found that the DCR of high-ADR patients had better efficacy than the low-ADR group of patients. The same trend was observed in the ORR; however, statistical significance was not reached (*P* > 0.05), which could be due to the small sample size. In survival analysis, the prognosis of low-ADR patients was significantly poorer than that of high-ADR patients. Even in other subgroups, with the exception of peritoneal metastasis, the results were still significant. In the peritoneal metastasis subgroup, it should be noted that the PFS difference between the two groups failed to achieve statistical significance, probably due to the study's small sample size (*n* = 75). Finally, in multivariable analysis, ADR was found to be an independent predictive factor for PFS and OS in patients with advanced GC.

Similar findings have been reported in other studies. One of these studies demonstrated that combined D-dimer and albumin levels may be used as prognostic markers for esophageal squamous cell carcinoma and nasopharyngeal carcinoma [23, 24]. Despite these findings, the mechanisms that explain this association in advanced GC are complex.

Among these mechanisms, the role of inflammation in tumorigenesis has been widely studied. Inflammatory conditions can lead to increased expression of inflammatory cytokines, including interleukin-6, TNF, and IL-1*β*, which increase the risk of cancer [35]. Specifically, inflammation plays a key role in the development, invasion, and metastasis of tumors, wherein the inflammatory microenvironment that is rich in immune cells, chemokines, and cytokine infiltrates surrounds the tumor and promotes malignant cell growth. These substances are produced by tumor cells or their surrounding tissues, leading to malignant progression [36]. During tumor development, inflammation may also contribute to tumor proliferation and metastasis by inhibiting apoptosis and promoting angiogenesis [37]. In addition, hyperinflammation persistence has been reported to be closely related to the outbreak of postoperative infectious complications, which also increase the risk of cancer [38]. Systemic inflammation also results in cancer cachexia syndrome, which is characterized by weight loss, anorexia, and fatigue, and can affect the quality of life and prognosis of patients [7]. Moreover, cancer-related inflammation can lead to changes in drug metabolism pathways and drug transporters, leading to slower clearance of anticancer drugs and increased treatment-related toxicity [7]. A previous study reported that high serum proinflammatory cytokine levels, such as interleukin-6, are associated with poor prognosis in human GC; thus, blocking these inflammatory responses may improve the prognosis of patients with tumors [7]. Furthermore, it has been reported that D-dimer is significantly increased in inflammatory diseases, and its level is negatively correlated with a good prognosis [33, 39]. In relation to this, inflammation also inhibits albumin synthesis, resulting in decreased albumin levels [40].

Aside from inflammation, the relationship between poor nutritional status and tumors also deserves attention [41]. Unlike other diseases, GC patients usually have a higher nutritional risk due to persistent gastric outlet obstruction, dietary restriction, malabsorption, chronic blood loss, and tumor consumption [42, 43]. A previous study has shown that 64.9% of GC patients have a Nutritional Risk Screening 2002 (NRS 2002) score of no less than 3, whereas for patients who underwent palliative surgery, the proportion of patients with NRS 2002 ≥ 3 increased to 68.6% [26]. Nutritional risk has been used as a predictor of postoperative mortality and complications in patients with gastrointestinal cancer [44, 45]. In relation to this, the nutritional status of cancer patients has a serious impact on the prognosis and quality of life (QOL). With the development of cancer, many patients lose their appetite and weight, falling into a state of malnutrition. Specifically, malnutrition may weaken the body's defense mechanisms, such as humoral and cellular immunity, leading to an increased likelihood of infection and poor response to anticancer treatment [46, 47]. Excessive surgical stress and postoperative complications can also lead to a systemic inflammatory response, which promotes malnutrition and tumor progression, resulting in a poor prognosis of cancer patients [48–50]. As a response, advanced GC patients have been reported to benefit from nutritional improvement following nutritional support [51]. In such cases, nutritionally supported albumin can stabilize cell growth and DNA replication, buffer various biochemical changes, and play an antioxidant role in carcinogens [52]. Thus, albumin is the most abundant serum protein and a useful biomedical factor for determining the nutritional status of patients.

Impaired fibrinolysis and coagulation systems are another hallmark of cancer since their components may promote the proliferation, survival, and angiogenesis of tumor cells [53]. Notably, coagulation-related molecules, such as fibrinogen and D-dimer, play roles in GC growth and progression, wherein their levels have been proposed as biomarkers to predict prognosis, treatment response, and thrombosis risk [30]. Cancer cells adversely interfere with normal cellular functions by expressing a variety of cytokines and proteins, which disrupt the balance between fibrinolysis and anticoagulation, lead to vascular endothelial injury and cytokine and agglutinant release, and promote tumor cell migration and invasion and tumor vasculature leakage [54]. Thus, anticoagulants play an important role in tumor treatment [53]. Among the fibrinolytic and coagulation factors in the tumor microenvironment, fibrinogen and D-dimer are the main components involved in multiple stages of tumor development. In particular, D-dimer, as a stable fibrin degradation product which can hint at abnormal fibrinolytic and coagulation activation, was a prognostic factor for multiple tumors, including GC [19, 22, 55, 56].

As mentioned above, cancer patients often have poor nutritional status, chronic inflammation, and a hypercoagulable state. Reduced albumin levels suggest systemic inflammation and severe nutritional status, whereas elevated D-dimer levels are correlated with excessive inflammatory reactions and coagulation abnormalities. In the present study, the combination of albumin and D-dimer, as compared to albumin and D-dimer alone, enabled ADR to be an improved predictor of survival outcome in advanced GC patients, demonstrating that decreased albumin and increased D-dimer levels simultaneously reflect the nature of the disease.

Our study had several advantages as compared to previous studies. First, this is the first report on pretreatment ADR's value in predicting the survival and effect of first-line chemotherapy in advanced GC patients. Second, the findings of this study showed that ADR had a higher AUC value than albumin and D-dimer, which may be due to ADR being a combination of these predictors of inflammatory, nutritional, and coagulation status of patients, enhancing its prognostic value. In fact, this is the first report of ADR as a superior predictor of OS compared to albumin and D-dimer alone. Moreover, ADR can still be used as a prognostic predictor of advanced GC in subgroups of normal albumin and D-dimer levels. These findings will help clinicians focus on patients who are easily ignored due to their normal blood tests and can help easily predict the treatment response of patients by objectively calculating the ADR value for individualized treatment adjustments. Finally, ADR, as a prognostic predictor in GC patients, is reasonably cheap, readily available, reproducible, and powerful.

Despite these findings, this study had several limitations. First, this was a retrospective, single-center study. Therefore, a prospective study design is required for further evaluation of our findings. Second, this study had a small sample size; thus, multicenter studies could help increase the sample size for further investigations on this matter.

## 5. Conclusions

Low-ADR GC patients have a lower DCR, shorter OS, and shorter PFS than high-ADR patients. Pretreatment ADR may also be a useful marker for chemotherapy efficacy and prognosis in advanced GC patients who receive first-line chemotherapy.

## Figures and Tables

**Table 1:** Relationship between the pretreatment ADR and clinicopathological factors.

	Total	Low-ADR	High-ADR	*P* value
*Total (n)*	247	86	161	
*Age (years)*	59 (52–64)	58 (51–62)	60 (52–65)	0.102
*Sex (n)*				
Male	163 (66.0%)	56 (65.1%)	107 (66.5%)	0.832
Female	84 (34.0%)	30 (34.9%)	54 (33.5%)	
Body mass index (kg/m^2^)	21.51 (19.57–23.43)	20.76 (19.34–23.63)	21.97 (19.79–23.27)	0.565

ECOG *PS score (n)*				
0-1	209 (84.6%)	73 (84.9%)	136 (84.5%)	0.932
≥2	38 (15.4%)	13 (15.1%)	25 (15.5%)	

*Pathological differentiation (n)*				
Well	67 (27.1%)	15 (17.4%)	52 (32.3%)	0.012
Poorly	180 (72.9%)	71 (82.6%)	109 (67.7%)	

*The number of organs affected by metastasis (n)*				
0-1	160 (64.8%)	50 (58.1%)	110 (68.3%)	0.110
≥2	87 (35.2%)	36 (41.9%)	51 (31.7%)	

*Peritoneal metastasis (n)*				
Yes	75 (30.4%)	35 (40.7%)	40 (24.8%)	0.010
No	172 (69.6%)	51 (59.3%)	121 (75.2%)	

*TNM stage (n)*				
III	51 (20.6%)	11 (12.8%)	40 (24.8%)	0.026
IV	196 (79.4%)	75 (87.2%)	121 (75.2%)	
Thrombocyte counts (×109/L)	257 (193–341)	269 (210–350)	247 (184–325)	0.031
Hemoglobin (g/L)	123.0 (106.0–139.0)	120.5 (104.5–134.3)	125.0 (106.5–139.0)	0.083
CEA (ng/mL)	3.60 (1.55–11.31)	4.66 (1.73–18.60)	2.97 (1.52–9.24)	0.173
CA19-9 (U/mL)	19.60 (7.94–111.90)	20.86 (8.79–124.53)	17.12 (7.84–95.39)	0.446
CA72-4 (U/mL)	7.52 (2.55–26.53)	12.13 (4.28–45.69)	5.19 (2.19–21.35)	0.001

ADR, albumin to D-dimer ratio; ECOG PS, Eastern Cooperative Oncology Group performance status; CEA, carcinoembryonic antigen; CA72-4, carbohydrate antigen 72-4; CA19-9, carbohydrate antigen 19-9.

**Table 2:** Correlations between PFS and patient and tumor characteristics.

	Univariate analysis		*P* value	Multivariate analysis		*P* value
	Hazard ratio	95% CI		Hazard ratio	95% CI	
Age (>59 years)	0.911	0.709–1.171	0.467			
Sex (male)	1.051	0.806–1.370	0.715			
Body mass index (<18.5 or >25 kg/m^2^)	0.971	0.731–1.292	0.842			
ECOG PS score (>1)	0.929	0.657–1.313	0.675			
Pathological differentiation (poorly)	1.197	0.902–1.590	0.214			
The number of organs affected by metastasis (>1)	1.511	1.161–1.967	0.002	1.299	0.954–1.768	0.096
Peritoneal metastasis (Yes)	1.506	1.144–1.983	0.004	1.147	0.822–1.602	0.419
TNM staging (IV)	1.375	1.009–1.875	0.044	1.017	0.720–1.436	0.924
CEA (>5 ng/mL)	1.240	0.959–1.604	0.101	1.134	0.857–1.501	0.377
CA19-9 (>37 U/mL)	0.925	0.713–1.200	0.558			
CA72-4 (>6 U/mL)	1.479	1.143–1.914	0.003	1.241	0.941–1.636	0.126
Thrombocyte counts (>300 × 10^9^/L)	1.100	0.843–1.435	0.483			
ADR (≥41.64)	0.445	0.338–0.585	<0.001	0.509	0.380–0.681	<0.001

ADR, albumin to D-dimer ratio; ECOG PS, eastern cooperative oncology group performance status; CEA, carcinoembryonic antigen; CA72-4, carbohydrate antigen 72-4; CA19-9, carbohydrate antigen 19-9; CI, confidence interval; PFS, progressive-free survival.

**Table 3:** Correlations between OS and patient and tumor characteristics.

	Univariate analysis		*P* value	Multivariate analysis		*P* value
	Hazard ratio	95% CI		Hazard ratio	95% CI	
Age (>59 years)	0.691	0.537–0.891	0.004	0.883	0.674–1.158	0.370
Sex (male)	1.090	0.836–1.420	0.525			
Body mass index (<18.5 or >25 kg/m^2^)	1.168	0.879–1.552	0.285			
ECOG PS score (>1)	0.951	0.671–1.349	0.780			
Pathological differentiation (poorly)	1.409	1.062–1.868	0.017	1.268	0.952–1.688	0.105
The number of organs affected by metastasis (>1)	1.493	1.146–1.946	0.003	1.272	0.933–1.733	0.128
Peritoneal metastasis (Yes)	1.924	1.457–2.542	<0.001	1.558	1.106–2.196	0.011
TNM staging (IV)	1.338	0.982–1.822	0.065	0.945	0.668–1.337	0.749
CEA (>5 ng/mL)	1.240	0.960–1.602	0.099	1.284	0.956–1.723	0.097
CA19-9 (>37 U/mL)	1.196	0.922–1.550	0.178	1.215	0.925–1.596	0.161
CA72-4 (>6 U/mL)	1.278	0.993–1.645	0.057	1.034	0.789–1.356	0.808
Thrombocyte counts (>300 × 10^9^/L)	1.164	0.891–1.520	0.267			
ADR (≥41.64)	0.273	0.202–0.369	<0.001	0.317	0.231–0.437	<0.001

ADR, albumin to D-dimer ratio; ECOG PS, Eastern Cooperative Oncology Group performance status; CEA, carcinoembryonic antigen; CA72-4, carbohydrate antigen 72-4; CA19-9, carbohydrate antigen 19-9; CI, confidence interval; OS, overall survival.

## Data Availability

The data used to support the findings of this study are included within the article.

## References

[1] Fitzmaurice C., Abate D., Abbasi N. (2019). Global, regional, and national cancer incidence, mortality, years of life lost, years lived with disability, and disability-adjusted life-years for 29 cancer groups, 1990 to 2017: a systematic analysis for the global burden of disease study. *JAMA Oncology*.

[2] Ajani J. A., Lee J., Sano T., Janjigian Y. Y., Fan D., Song S. (2017). Gastric adenocarcinoma. *Nature Reviews Disease Primers*.

[3] Wagner A. D., Syn N. L., Moehler M. (2017). Chemotherapy for advanced gastric cancer. *The Cochrane Database of Systematic Reviews*.

[4] Japanese Gastric Cancer Association (2017). Japanese gastric cancer treatment guidelines 2014 (ver. 4). *Gastric Cancer*.

[5] Mantovani A. (2009). Inflaming metastasis. *Nature*.

[6] Hébuterne X., Lemarié E., Michallet M., de Montreuil C. B., Schneider S. M., Goldwasser F. (2014). Prevalence of malnutrition and current use of nutrition support in patients with cancer. *Journal of Parenteral and Enteral Nutrition*.

[7] Diakos C. I., Charles K. A., McMillan D. C., Clarke S. J. (2014). Cancer-related inflammation and treatment effectiveness. *The Lancet Oncology*.

[8] Mantovani A., Allavena P., Sica A., Balkwill F. (2008). Cancer-related inflammation. *Nature*.

[9] Kim A. J., Hong D. S., George G. C. (2021). Diet-related interventions for cancer-associated cachexia. *Journal of Cancer Research and Clinical Oncology*.

[10] Zhang L., Wang Z., Xiao J. (2020). Sodium to globulin ratio as a prognostic factor for patients with advanced gastric cancer. *Journal of Cancer*.

[11] Quispe E. Á., Li X.-M., Yi H. (2016). Comparison and relationship of thyroid hormones, IL-6, IL-10 and albumin as mortality predictors in case-mix critically ill patients. *Cytokine*.

[12] Kapoor A., Dhandapani S., Gaudihalli S., Dhandapani M., Singh H., Mukherjee K. K. (2018). Serum albumin level in spontaneous subarachnoid haemorrhage: more than a mere nutritional marker!. *British Journal of Neurosurgery*.

[13] Antkowiak M., Gabr A., Das A. (2019). Prognostic role of albumin, bilirubin, and ALBI scores: analysis of 1000 patients with hepatocellular carcinoma undergoing radioembolization. *Cancers*.

[14] Man Y.-N., Chen Y.-F. (2019). Systemic immune-inflammation index, serum albumin, and fibrinogen impact prognosis in castration-resistant prostate cancer patients treated with first-line docetaxel. *International Urology and Nephrology*.

[15] Filliatre-Clement L., Broseus J., Muller M. (2019). Serum albumin or body mass index: which prognostic factor for survival in patients with acute myeloblastic leukaemia?. *Hematological Oncology*.

[16] Oh S. E., Choi M.-G., Seo J.-M. (2019). Prognostic significance of perioperative nutritional parameters in patients with gastric cancer. *Clinical Nutrition*.

[17] Wojtukiewicz M. Z., Hempel D., Sierko E., Tucker S. C., Honn K. V. (2016). Thrombin-unique coagulation system protein with multifaceted impacts on cancer and metastasis. *Cancer and Metastasis Reviews*.

[18] Weitz J. I., Fredenburgh J. C., Eikelboom J. W. (2017). A test in context: D-dimer. *Journal of the American College of Cardiology*.

[19] Watanabe A., Araki K., Harimoto N. (2018). D-dimer predicts postoperative recurrence and prognosis in patients with liver metastasis of colorectal cancer. *International Journal of Clinical Oncology*.

[20] Chen X., Chang Z., Liu Z. (2019). D-dimer increase: an unfavorable factor for patients with primary liver cancer treated with TACE. *Cancer Chemotherapy and Pharmacology*.

[21] Deng H.-Y., Zheng X., Jiang R., Wang R.-L., Zhou J., Qiu X.-M. (2019). Preoperative D-dimer level is an independent prognostic factor for non-small cell lung cancer after surgical resection: a systematic review and meta-analysis. *Annals of Translational Medicine*.

[22] Hara K., Aoyama T., Hayashi T. (2020). Postoperative D-dimer elevation affects tumor recurrence and the long-term survival in gastric cancer patients who undergo gastrectomy. *International Journal of Clinical Oncology*.

[23] Liu D.-Q., Li F.-F., Jia W.-H. (2016). Cumulative scores based on plasma D-dimer and serum albumin levels predict survival in esophageal squamous cell carcinoma patients treated with transthoracic esophagectomy. *Chinese Journal of Cancer*.

[24] He S.-S., Wang Y., Wang C.-T. (2019). A combined marker based on plasma D-dimer and serum albumin levels in patients with nasopharyngeal carcinoma is associated with poor survival outcomes in a retrospective cohort study. *Journal of Cancer*.

[25] Piazuelo M. B., Riechelmann R. P., Wilson K. T., Algood H. M. S. (2019). Resolution of gastric cancer-promoting inflammation: a novel strategy for anti-cancer therapy. *Current Topics in Microbiology and Immunology*.

[26] Li Y.-F., Nie R.-C., Wu T. (2019). Prognostic value of the nutritional risk screening 2002 scale in metastatic gastric cancer: a large-scale cohort study. *Journal of Cancer*.

[27] Kanda M., Tanaka C., Kobayashi D. (2017). Proposal of the coagulation score as a predictor for short-term and long-term outcomes of patients with resectable gastric cancer. *Annals of Surgical Oncology*.

[28] Ock C.-Y., Nam A.-R., Bang J.-H. (2017). Signature of cytokines and angiogenic factors (CAFs) defines a clinically distinct subgroup of gastric cancer. *Gastric Cancer*.

[29] Hirahara N., Tajima Y., Fujii Y. (2018). Prognostic nutritional index as a predictor of survival in resectable gastric cancer patients with normal preoperative serum carcinoembryonic antigen levels: a propensity score matching analysis. *BMC Cancer*.

[30] Repetto O., De Re V. (2017). Coagulation and fibrinolysis in gastric cancer. *Annals of the New York Academy of Sciences*.

[31] Eckart A., Struja T., Kutz A. (2020). Relationship of nutritional status, inflammation, and serum albumin levels during acute illness: a prospective study. *The American Journal of Medicine*.

[32] Gupta D., Lis C. G. (2010). Pretreatment serum albumin as a predictor of cancer survival: a systematic review of the epidemiological literature. *Nutrition Journal*.

[33] Zhang W., Sang L., Shi J. (2021). Association of D-dimer elevation with inflammation and organ dysfunction in ICU patients with COVID-19 in Wuhan, China: a retrospective observational study. *Aging*.

[34] Lin Y., Liu Z., Qiu Y. (2018). Clinical significance of plasma D-dimer and fibrinogen in digestive cancer: a systematic review and meta-analysis. *European Journal of Surgical Oncology*.

[35] Zhong Z., Sanchez-Lopez E., Karin M. (2016). Autophagy, inflammation, and immunity: a troika governing cancer and its treatment. *Cell*.

[36] Grivennikov S. I., Greten F. R., Karin M. (2010). Immunity, inflammation, and cancer. *Cell*.

[37] Khandia R., Munjal A. (2020). Interplay between inflammation and cancer. *Inflammatory Disorders, Part A*.

[38] Kamonvarapitak T., Matsuda A., Matsumoto S. (2020). Preoperative lymphocyte-to-monocyte ratio predicts postoperative infectious complications after laparoscopic colorectal cancer surgery. *International Journal of Clinical Oncology*.

[39] Semeraro F., Ammollo C. T., Caironi P. (2020). D-dimer corrected for thrombin and plasmin generation is a strong predictor of mortality in patients with sepsis. *Blood Transfusion = Trasfusione Del Sangue*.

[40] van Gelder M. K., Abrahams A. C., Joles J. A., Kaysen G. A., Gerritsen K. G. F. (2018). Albumin handling in different hemodialysis modalities. *Nephrology Dialysis Transplantation*.

[41] Barreira J. V. (2020). The role of nutrition in cancer patients. *Nutrition and Cancer*.

[42] Zheng H.-L., Lu J., Li P. (2017). Effects of preoperative malnutrition on short- and long-term outcomes of patients with gastric cancer: can we do better?. *Annals of Surgical Oncology*.

[43] Lim H. S., Lee B., Cho I., Cho G. S. (2020). Nutritional and clinical factors affecting weight and fat-free mass loss after gastrectomy in patients with gastric cancer. *Nutrients*.

[44] Zhao Y., Deng Y., Peng J. (2018). Does the preoperative prognostic nutritional index predict survival in patients with liver metastases from colorectal cancer who underwent curative resection?. *Journal of Cancer*.

[45] Kuroda D., Sawayama H., Kurashige J. (2018). Controlling nutritional status (CONUT) score is a prognostic marker for gastric cancer patients after curative resection. *Gastric Cancer*.

[46] Maggini S., Pierre A., Calder P. C. (2018). Immune function and micronutrient requirements change over the life course. *Nutrients*.

[47] Villena J., Shimosato T., Vizoso-Pinto M. G., Kitazawa H. (2020). Editorial: nutrition, immunity and viral infections. *Frontiers in Nutrition*.

[48] Migita K., Matsumoto S., Wakatsuki K. (2019). Postoperative serum C-reactive protein level predicts long-term outcomes in stage I gastric cancer. *Journal of Surgical Research*.

[49] Matsubara D., Arita T., Nakanishi M. (2020). The impact of postoperative inflammation on recurrence in patients with colorectal cancer. *International Journal of Clinical Oncology*.

[50] Shibutani M., En W., Okazaki Y., Maeda K., Hirakawa K., Ohira M. (2021). A high postoperative serum C-reactive protein level has a negative impact on long-term survival, regardless of postoperative infectious complications, in patients who undergo laparoscopic surgery for colorectal cancer. *Anticancer Research*.

[51] Lu Z., Fang Y., Liu C. (2021). Early interdisciplinary supportive care in patients with previously untreated metastatic esophagogastric cancer: a phase III randomized controlled trial. *Journal of Clinical Oncology*.

[52] Arroyo V., García-Martinez R., Salvatella X. (2014). Human serum albumin, systemic inflammation, and cirrhosis. *Journal of Hepatology*.

[53] Kwaan H. C., Lindholm P. F. (2019). Fibrin and fibrinolysis in cancer. *Seminars in Thrombosis and Hemostasis*.

[54] Bikdeli B., Sharif-Kashani B., Chitsaz E. (2011). Dexter versus sinister deep vein thrombosis: which is the more sinister? findings from the NRITLD DVT registry. *Seminars in Thrombosis and Hemostasis*.

[55] Watanabe A., Harimoto N., Araki K. (2020). D-dimer could be a surrogate postoperative prognostic marker of resectable pancreatic cancer. *Pancreas*.

[56] Shiina Y., Nakajima T., Yamamoto T. (2019). The D-dimer level predicts the postoperative prognosis in patients with non-small cell lung cancer. *PLoS One*.

